# Genetic contribution of caspase-8 variants and haplotypes to breast cancer risk and prognosis: a case-control study in Iran

**DOI:** 10.1186/s12920-023-01484-0

**Published:** 2023-04-04

**Authors:** Fahimeh Afzaljavan, Elham Vahednia, Matineh Barati Bagherabad, Fatemeh Vakili, Atefeh Moezzi, Azar Hosseini, Fatemeh Homaei Shandiz, Mohammad Mahdi Kooshyar, Mohammadreza Nassiri, Alireza Pasdar

**Affiliations:** 1 Department of Medical Genetics and Molecular Medicine, Faculty of Medicine, Mashhad University of Medical Sciences, Mashhad, Iran; 2 Midwifery department, Faculty of Nursing and Midwifery, Mashhad University of Medical Sciences, Mashhad, Iran; 3 Pharmacological Research Center of Medicinal Plants, Mashhad University of Medical Sciences, Mashhad, Iran; 4 Cancer research center, Mashhad University of Medical Sciences, Mashhad, Iran; 5 Department of Internal Medicine, Faculty of Medicine, Ghaem Medical Center, Mashhad University of Medical sciences, Mashhad, Iran; 6 Recombinant Protein Research Group, The Research Institute of Biotechnology, Ferdowsi University of Mashhad, Mashhad, Iran; 7 Bioinformatics Research Centre, Mashhad University of Medical Sciences, Mashhad, Iran

**Keywords:** Breast neoplasm, Biomarker, Caspase 8, Diplotype, Overall survival, Prognosis

## Abstract

**Purpose:**

Multiple genome-wide and candidate-gene association studies have been conducted to search for common risk variants of breast cancer. Recent large meta-analyses and consolidating evidence have highlighted the role of the caspase-8 gene in breast cancer pathogenesis. Therefore, this study aimed to identify common variations and haplotypes associated with risk and overall survival of breast cancer with respect to underlying susceptibility variants in the *CASP8* gene region in a group of the Iranian population.

**Methods:**

In a case-control study with a total of 1008 samples (455 cases and 553 controls), genotyping of 12 candidate polymorphisms, consisting of *rs3834129*, *rs2037815*, *rs7608692*, *rs12990906*, *rs3769821*, *rs6435074*, *rs3754934*, *rs3817578*, *rs10931936*, *rs1045485*, *rs1045487,* and *rs13113*, were performed using PCR-based methods, including ARMS-PCR, AS-PCR, RFLP-PCR, HRM-PCR, and TaqMan-PCR.

**Results:**

*rs3834129*, *rs3754934*, *rs12990906*, and *rs10931936* were associated with the risk and overall survival of breast cancer. Several haplotypes were also identified an associated with a higher risk of breast cancer, including a three-SNP haplotype *rs3817578*-*rs10931936*-*rs1045485* [*p* < 0.001, OR = 1.78(1.32–2.41)]. *rs3754934-*C allele showed an association with a lower risk of death in all patients [*p* = 0.022; HR = 0.46(0.23–0.89)] and in the hormone-receptor-positive group [*p* = 0.038; HR = 0.37(0.14–0.95)], as well as CC genotype in the hormone-receptor-positive group [*p* = 0.002; HR = 0.09(0.02–0.43)].

**Conclusion:**

The present study suggests a diagnostic and prognostic role of *CASP8* gene variations in breast cancer. The risky haplotypes are likely to have one or more underlying breast cancer susceptibility alleles. Understanding the mode of action of these alleles will aid individual-level risk prediction. It also may help identify at-risk patients to provide them with better surveillance.

**Supplementary Information:**

The online version contains supplementary material available at 10.1186/s12920-023-01484-0.

## Introduction

Breast cancer is the most common cancer among women, accounting for 11.7% of all new cancers and 24.5% of all female cancers. Moreover, it is the fifth cause of cancer death and first-ranked in women [1]. Epidemiological studies indicated that progression in detection methods like mammographic screening led to increasing incidence rates of breast cancer during the 1980-90s decades in many countries. Conversely, widespread screening and reduced menopausal hormone therapy caused a decreased incidence during the early 2000s. However, breast cancer incidence is rising due to changes in lifestyle, sociocultural, and environmental issues. High Body Mass Index (BMI) resulting from a sedentary lifestyle and junk and high-calorie diet, night shift, and reproductive and gynecologic factors, including hormonal changes, reduced pregnancy, and lactation, have been identified as the risk factors of the disease. Therefore, identifying diagnostic and prognostic markers of the disease is a prominent point of attention in oncology research [2].

It is estimated that 5–10% of breast cancers are hereditary; however, a high portion of the disease is sporadic type affected by genetic and environmental risk factors, although most of the underlying genetic mechanisms have not been fully defined [3]. Among genetic indicators, polymorphisms are common genomic variations in the general population identified as potential genetic markers for risk assessment. However, comparing high penetrance mutations, these are typically associated with moderate risk [4]. Although candidate gene studies have introduced various loci [5], in recent years, high-throughput genome-wide association studies have identified many genetic loci associated with the risk of breast cancer, introducing breast cancer as a polygenic complex disease [6].

Caspase 8 protein (CASP8), a 55 kDa cysteine protease, is a member of the caspase family and a key apoptosis signaling molecule. It contributes to inducing cell death, particularly through the death receptor pathway. *CASP8*, one of the first low penetrance loci, has been identified to be associated with the risk of breast cancer in candidate gene studies [7–10]. Furthermore, efforts to identify new variations in fine-mapping [11, 12] and genome-wide association [13] studies have provided evidence of the association of several variants of *CASP8* with breast cancer risk. Given the importance of allelic variations associated with cancers, including breast cancer [7–13], this study aimed to investigate the association of *CASP8* polymorphisms, haplotypes and diplotypes with breast cancer risk, prognosis, and clinicopathological features in a northeastern population of Iran.

## Materials and methods

### Study population

This study was approved by the Ethics Committee of Mashhad University of Medical Sciences under the ethical approval number: IR.MUMS.REC.1394.188. All participants signed a written informed consent at the time of study entry.

Due to the fact that *CASP8* had not been assessed in previous research in Iran, we did not have access to the allele frequency of its variation in our population to calculate the exact sample size required for a decent power of the study (80%). Consequently, a pilot sample size was performed based on similar studies in this field, which mainly have suggested 200–400 samples in each group. However, the final study population included 1008 participants. The breast cancer group included 455 patients (152 new cases diagnosed between 2016 and 2018 and 303 patients diagnosed between 1987 and 2016 and followed in this period) referred to academic teaching hospitals of Mashhad University of Medical Sciences. The control group consisted of 553 healthy people referred to clinicians between 2016 and 2018 for screening, and their health was confirmed using the clinical breast exam (CBE) and mammography. Demographic information was collected using a questionnaire providing sociodemographic data, including age, age of menarche, menopause and first gestation, BMI, history of lactation and abortion, and physical activity.

Pedigree was drawn for all participants to check the family history of cancer and find participants’ relatives. Manchester Score (MS) was used to identify the probability of harboring *BRCA1*/*2* mutations [14]. As a result, the highly suspected hereditary cancer was excluded. After excluding five patients with probable hereditary breast cancer (with an MS of more than 10), 450 sporadic cancer subjects entered the study as the patient group.

The histopathological data, including breast tumor subtype, stage, grade, and receptor status (ER, PR, and HER2), was extracted from patients’ medical records. Categorization was performed according to the standard protocols of the world health organization (WHO) [15], the American Joint Committee on Cancer (AJCC) [16], and the American Society of Clinical Oncology (ASCO) [17]. All cases were followed, and new events, including recurrence, secondary tumors, and metastasis, were documented.

### Blood collection and DNA extraction

Five ml of peripheral blood was collected using a Vacuette K2-EDTA blood collection tube (Greiner Bio-One, USA). The salting-out method was utilized to isolate DNA [18]. The qualification and quantification of extracted DNA were evaluated by gel electrophoresis and Epoch™ Microplate Spectrophotometer (BioTek Instruments Inc., Winooski, VT, USA). Samples were aliquoted in a concentration of 150 ng per microliter and stored at -20 until polymerase chain reaction (PCR) analysis.

### SNP selection

Twelve validated polymorphisms of the *CASP8* gene were selected in different gene regions, including 5’ UTR (promoter), exon, intron, and 3’ UTR regions. Selection of polymorphisms was performed based on several criteria, including validation of the association in numerous GWAS studies, which denotes a strong association with breast cancer risk in different populations. We also considered selecting SNPs that are located in the same region to be able to perform haplotype analysis to examine the overall effect of these polymorphisms. We also considered selecting markers with an acceptable MAF and heterozygosity (minor allele frequency > 5% and heterozygosity > 10%) to achieve the highest possible study power. Characteristics of the selected polymorphisms have been shown in (Additional file 1: Supplementary Table 1).

### Genotyping

Genotyping was done using different PCR-based methods. *rs3834129*, *rs12990906*, rs3754934, *rs3817578* ,and *rs10931936* were genotyped using Tetra-ARMS-PCR, *rs2037815* and *rs7608692* using allele-specific PCR, *rs3769821* and *rs1045485* using RFLP-PCR. Genotyping method for *rs1045487* and *rs6435074* was HRM (LightCycler® 96 Instrument (Roche Molecular Systems, Inc.)), and for *rs13113* was TaqMan (SNP genotyping Assays (TaqMan®), Catalog number: 4,351,379; Rotor-Gene 6000™ real-time analyzer (Applied Biosystems)). Primers were designed using Primer1, Gene runner and WASP (Web-based Allele-Specific PCR assay), and evaluated using Oligoanalyzer and Mfold. The designed primer sequences have been shown in (Additional file 1: Supplementary Table 2).

Amplification reactions and protocols are shown in (Additional file 1: Supplementary Tables 3 & 4). 5% of samples were randomly re-genotyped to verify genotyping results for quality control purposes. In addition, three samples were randomly sanger sequenced to validate the genotyping method for each marker. Sequencing was done using outer primers for polymorphisms genotyped by Tetra-ARMS-PCR, and new primers, outer both sides of the genotyped region, were designed for the other variations.

### Haplotype and diplotype analysis

Assessing the haplotypes and diplotypes distribution was carried out using the PHASE software version 2.1.1 for windows [19]. The linkage disequilibrium (LD) was calculated by 2LD program version 1.00 and evaluated by the D′ statistic as the deviation between the expected haplotype and observed frequency [20].

### Statistical analysis

The Hardy-Weinberg Equilibrium (HWE) assumption was assessed in the case and control samples using the *χ*^*2*^ with one degree of freedom. Data are shown in (Additional file 1: Supplementary Table 5). Depending on the assessment of normality using the Kolmogorov-Smirnov (K-S) test, the normally distributed continuous variables were examined using the independent sample t-test and the Mann-Whitney U test was used to compare non-normally distributed variables between the two groups. ANOVA or Kruskal Wallis was also used to compare more than two groups. The categorical variables were compared appropriately with the chi-square or Fisher’s exact tests. Correlations between variables were tested using the Pearson correlation test for normally distributed variables and the Spearman correlation test for non-normally distributed variables.

The associations of alleles, genotypes, haplotypes, and diplotypes with breast cancer risk, breast cancer risk factors, and histopathological status were judged by logistic regression. Odds ratios (ORs) and 95% confidence intervals (CIs) were calculated for the measured risk factors. Multivariate logistic regression was applied to identify the variables with independent association with the risk of breast cancer. The backward logistic regression (LR) model was implemented to select variables for multivariable investigation. The results were also adjusted for potential confounders such as BMI, age at first gestation, and Menopause status in the logistic regression analysis.

Overall survival (OS) time was considered the time between diagnosis according to the first biopsy confirming the disease and the time of death due to cancer or last contact. Kaplan–Meier plots/Log-rank and Cox proportional hazards regression approaches were used to explain the associations between different covariates and overall survival. The hazard rate ratio (HR) and 95% CIs were calculated by the Cox models.

Statistical analysis was performed using SPSS 16.0 (IBM, USA), and a P-value less than 0.05 was considered significant.

## Results

### Characteristics of the population

After excluding 5 patients with hereditary breast cancer, 450 breast cancer patients (mean age = 47.20 ± 10.41) and 553 healthy individuals (mean age = 45.88 ± 11.51) were studied. The characteristics of breast cancer cases and cancer-free controls have been shown in Table 1. Furthermore, tumor features of breast cancer patients have been reported in Table 2.

**Table 1:** The characteristics of breast cancer cases and cancer-free controls

Characteristic ^a^		Breast cancer	Control	P-value ^b^	OR (95%CI)
**Age**		47.20 ± 10.41	45.88 ± 11.51	0.065	1.01 (0.99–1.02)
**Age of menarche**		13.05 ± 1.65	13.23 ± 1.56	0.116	1.07 (0.98–1.16)
**Age of menopause** ^**c**^		47.79 ± 5.61	48.19 ± 5.21	0.545	1.01 (0.97–1.06)
**Age of first gestation**		21.39 ± 5.09	22.55 ± 4.53	**0.001**	1.05 (1.02–1.08)
**BMI (Kg/m** ^**2**^ **)** ^**d**^		27.66 ± 5.04	25.36 ± 4.36	**< 0.001**	1.11 (1.08–1.14)
**BMI (Kg/m** ^**2**^ **)**	BMI < 25	117 (28.4%)	260 (50.4%)	Reference	
	BMI ≥ 25	295 (71.6%)	256 (49.6%)	**< 0.001**	2.56 (1.94–3.37)
**Menopause status**	Pri & pre	238 (57.9%)	397 (74.8%)	Reference	
	Post	173 (42.1%)	134 (25.2%)	**< 0.001**	2.15 (1.63–2.84)
**History of lactation**	Negative	18 (4.7%)	20 (4.9%)	Reference	
	Positive	362 (95.3%)	390 (95.1%)	0.926	1.03 (0.54–1.98)
**History of abortion**	Negative	236 (64.1%)	281 (70.3%)	Reference	
	Positive	132 (35.9%)	119 (29.8%)	0.071	1.32 (0.98–1.79)
**Physical activity**	Negative	125 (42.4%)	51 (13.3%)	Reference	
	Positive	170 (57.6%)	332 (86.7%)	**< 0.001**	4.66 (3.20–6.80)

^a^ Data are presented as mean ± SD for continuous variable or number (percentage, %) for categorical variables;

^b^ Significant data has been shown in bold

^c^ The age of menopause in individuals with natural menopause

^d^ BMI: Body Mass Index

**Table 2:** Distribution of tumour characteristics of Breast cancer cases

Characteristics		Number	Percent
**Tumor subtype**	Invasive Ductal Carcinoma	338	75.1
	Precursor lesions	19	4.2
	Invasive Lobular Carcinoma	11	2.4
	Invasive Medulary Carcinoma	7	1.6
	Metastatic Carcinoma	11	2.4
	Others	13	2.9
	Unreported	51	11.3
**Grade**	Low grade (I & II)	252	56
	High grade (III)	96	21.3
	Unreported	102	22.7
**Tumor size**	Small (I & II)	292	64.9
	Large (III & IV)	73	16.2
	Unreported	85	18.9
**Lymph node**	Negative	144	32
	Positive (I, II & III)	213	47.4
	Unreported	93	20.4
**Metastasis**	Negative	338	75.1
	Positive	22	4.9
	Unreported	90	20
**Stage**	Early stage (I & II)	128	50.7
	Late stage (III & IV)	130	28.9
	Unreported	92	20.4
**ER status** ^**a**^	Negative	101	22.4
	Positive	295	65.6
	Unreported	54	12
**PR status** ^**b**^	Negative	114	25.3
	Positive	281	62.4
	Unreported	55	12.2
**HER2** ^**c**^	Negative	257	57.1
	Positive	103	22.9
	Equivocal	25	5.6
	Unreported	65	14.4
**Receptor status**	ER/PR + HER2 +/-	305	67.7
	ER/PR - HER2 +	41	9.1
	Triple negative (TNBC)	45	10
	Unreported	59	13.1

^a^ ER; Oestrogen receptor;

^b^ PR; Progesterone receptor

^c^ HER2; Human Epidermal growth factor Receptor 2

Menstrual status was significantly different between the two groups (p < 0.001). According to the findings of this study, there was no significant difference in lactation and abortion history between the groups (p > 0.05). BMI showed a significant difference (p < 0.001) with a mean of 27.65 ± 5.05 Kg/m2 in patients and 25.36 ± 4.36 Kg/m2 in healthy subjects. Also, the classification of this index into two groups of less and more than 25 showed that the percentage of people with a BMI above 25 in the patient group was higher than in the control group (p < 0.001).

Evaluation of clinicopathologic features indicated the most common type of tumor in the study population was the invasive ductal type by 75.1% of the total specimens examined. In situ, lobular and metastatic tumors were less prevalent. Tumor grade and stage examination showed that more patients (56%) had low-grade tumors, and 50.7% of patients were identified in the early stages of the disease (1 and 2). In terms of tumor size, small tumors (with 64.9% of all specimens) ranked first. Findings related to lymph node status showed that 47.4% of patients were lymph node-positive, with the highest number of involved nodes being between 1 and 3. Assessment of hormone receptor status showed that in more than 60% of patients, estrogen or progesterone hormone receptors were positive, and HER2 overexpression was observed in 22.9% of patients.

Evaluation of overall survival in patients showed that 5-year overall survival was 90%, and 10-year overall survival was 85%.

### Association of *CASP8* genotypes, haplotypes and diplotypes with breast cancer risk

Hardy–Weinberg equilibrium in the healthy controls is shown in (Additional file 1: Supplementary Table 5). For those polymorphisms which were not in Hardy–Weinberg equilibrium the genotyping results were verified by regenotyping 5% of samples randomly and the results were consistent with the previously genotyped samples. The results of statistical analysis showed that *rs3834129* was associated with breast cancer risk in dominant (II + ID vs. DD) (*p*_*Adj*_=0.034) and recessive (ID + DD vs. II) (*p*_*Adj*_=0.014) models. In the dominant model, *rs2037815*-G allele carriers (GA + GG) (*p*_*Adj*_=0.031), *rs7608692*-A-allele carriers (GA + AA) (*p*_*Adj*_=0.006), and *rs10931936*-T allele carriers (TT + CT) (*p*_*Adj*_<0.001) had a higher risk of breast cancer. On the other hand, carriers of the *rs3754934*-A allele (CA + AA) had a reduced risk of breast cancer in the dominant model (*p*_*Adj*_=0.004). We did not find a significant association between breast cancer risk and *rs3769821*, *rs6435074*, *rs3817578*, *rs1045485*, *rs1045487,* and *rs13113* in our study population. Alleles and genotypes frequencies have been reported in Table 3, for further information about the analyses based on different genetic models see (Additional file 1: Supplementary Table 6), and significant findings have been shown in Tables 4 and 5.

**Table 3:** The frequency of alleles and genotypes of CASP8 polymorphisms in breast cancer and healthy groups

SNP ID	Genotype	Breast cancer	Control	P-value _Adj_. ^a^	OR (95%CI) _Adj_.
**rs3834129**	DD	58 (12.9%)	95 (17.2%)	Reference	
	ID	185 (41.1%)	261 (47.2%)	0.158	1.49 (0.86–2.59)
	II	207 (46.0%)	197 (35.6%)	**0.008**	2.14 (1.22–3.75)
	D	301 (33.4%)	451 (40.8%)	Reference	
	I	599 (66.6%)	655 (59.2%)	**0.011**	1.43 (1.15–1.78)
**rs2037815**	AA	80 (17.8%)	130 (23.5%)	Reference	
	GA	261 (58.0%)	301 (54.4%)	0.514	1.15 (0.75–1.75)
	GG	109 (24.2%)	122 (22.1%)	0.502	0.84 (0.51–1.39)
	A	421 (46.8%)	561 (50.7%)	Reference	
	G	479 (53.2%)	545 (49.3%)	0.684	1.04 (0.85–1.29)
**rs7608692**	GG	161 (35.8%)	249 (45.0%)	Reference	
	GA	211 (46.9%)	215 (38.9%)	**0.006**	1.52 (1.12–2.04)
	AA	78 (17.3%)	89 (16.1%)	0.136	1.35 (0.91-2.00)
	G	533 (59.2%)	713 (64.5%)	Reference	
	A	367 (40.8%)	393 (35.5%)	0.179	1.16 (0.93–1.44)
**rs12990906**	CC	69 (15.3%)	111 (20.1%)	Reference	
	TC	196 (43.6%)	248 (44.8%)	0.172	1.41 (0.86–2.30)
	TT	185 (41.1%)	194 (35.1%)	0.712	1.09 (0.69–1.73)
	C	334 (37.1%)	470 (42.5%)	Reference	
	T	566 (62.9%)	636 (57.5%)	**0.026**	1.27 (1.03–1.58)
**rs3769821**	TT	273 (60.7%)	361 (65.3%)	Reference	
	TC	147 (32.7%)	143 (25.9%)	0.389	1.18 (0.81–1.72)
	CC	30 (6.7%)	49 (8.9%)	0.92	1.03 (0.54–1.96)
	T	693 (77.0%)	865 (78.2%)	Reference	
	C	207 (23.0%)	241 (21.8%)	0.533	1.08 (0.84-1-39)
**rs6435074**	CC	227 (50.4%)	294 (53.2%)	Reference	
	CA	181 (40.2%)	218 (39.4%)	0.841	1.06 (0.57–1.98)
	AA	42 (9.3%)	41 (7.4%)	0.924	1.03 (0.55–1.93)
	C	635 (70.6%)	806 (72.9%)	Reference	
	A	265 (29.4%)	300 (27.1%)	0.856	1.02 (0.81–1.29)
**rs3754934**	CC	385 (85.6%)	464 (84.1%)	Reference	
	CA	51 (11.3%)	75 (13.6%)	**0.002**	0.41 (0.23–0.73)
	AA	14 (3.1%)	13 (2.4%)	0.856	0.89 (0.26–3.07)
	C	821 (91.2%)	1005 (91.9%)	Reference	
	A	79 (8.8%)	101 (8.1%)	0.051	1.50 (0.99–2.27)
**rs3817578**	TT	8 (1.8%)	16 (2.9%)	Reference	
	CT	119 (26.4%)	142 (25.7%)	0.555	0.89 (0.60–1.32)
	CC	323 (71.8%)	395 (71.4%)	0.88	0.91 (0.29–2.91)
	T	135 (15.0%)	174 (15.7%)	Reference	
	C	765 (85.0%)	932 (84.3%)	0.993	1.00 (0.74–1.35)
**rs10931936**	CC	245 (54.4%)	396 (71.6%)	Reference	
	CT	168 (37.3%)	122 (21.1%)	**< 0.001**	2.31 (1.57–3.39)
	TT	37 (8.2%)	35 (6.3%)	0.372	1.34 (0.70–2.65)
	C	658 (73.1%)	914 (82.6%)	Reference	
	T	242 (26.9%)	192 (17.4%)	**< 0.001**	1.73 (1.34–2.23)
**rs1045485**	CC	30 (6.7%)	56 (10.1%)	Reference	
	GC	127 (28.2%)	142 (25.7%)	0.744	1.07 (0.72–1.58)
	GG	293 (65.1%)	355 (64.2%)	0.28	0.69 (0.35–1.35)
	C	187 (20.8%)	254 (23.0%)	Reference	
	G	713 (79.2%)	852 (77.0%)	0.296	1.15 (0.88–1.45)
**rs1045487**	GG	321 (71.3%)	403 (72.9%)	Reference	
	GA	111 (24.7%)	135 (24.4%)	0.912	1.02 (0.68–1.53)
	AA	18 (4.0%)	15 (2.7%)	0.113	2.30 (0.82–6.44)
	G	753 (83.7%)	941 (85.1%)	Reference	
	A	147 (16.3%)	165 (14.9%)	0.15	1.24 (0.92–1.67)
**rs13113**	TT	170 (37.8%)	228 (41.2%)	Reference	
	TA	204 (45.3%)	250 (45.2%)	0.804	1.04 (0.72–1.52)
	AA	76 (16.9%)	75 (13.6%)	0.285	1.33 (0.79–2.24)
	T	544 (60.4%)	706 (63.8%)	Reference	
	A	356 (39.6%)	400 (36.2%)	0.133	1.18 (0.95–1.46)

^a^ significant data has been shown in bold

**Table 4:** Association of CASP8 polymorphism, haplotypes and diplotypes with breast cancer risk, the clinico-pathological features and overall survival

Characteristics	Polymorphism/ Haplotype/ Diplotype	P-value _Adj_. ^a^	OR/HR (95%CI) _Adj_	D’
Breast cancer risk	*rs3834129* (II + ID vs. DD)	0.034	1.76 (1.04–2.97)	
Breast cancer risk	*rs3834129* (ID + DD vs. II)	0.014	1.58 (1.09–2.67)	
Breast cancer risk	*rs2037815* (GA + GG vs. AA)	0.031	1.44 (1.03–2.01)	
Breast cancer risk	*rs7608692* (GA + AA vs. GG)	0.006	1.47 (1.12–1.93)	
Breast cancer risk	*rs3754934* (CA + AA vs. CC)	0.004	0.49 (0.27–0.78)	
Breast cancer risk	*rs10931936* (CT + TT vs. CC)	< 0.001	2.06 (1.44–2.93)	
Breast cancer risk	Haplotype *rs3817578*-*rs10931936*- *rs1045485* (CTG vs. Others)	< 0.001	1.78 (1.32–2.41)	0.52
Breast cancer risk	Haplotype *rs3754934*-*rs3817578*-*rs10931936*-*rs1045485* (CCTG vs. Others)	< 0.001	1.75 (1.30–2.38)	0.61
Breast cancer risk	Haplotype *rs3754934*-*rs3817578*-*rs10931936*-*rs1045485* (ATCG vs. Others)	0.007	0.43 (0.24–0.79)	0.61
Breast cancer risk	Haplotype *rs3754934*-*rs381757*-*rs10931936*-*rs1045485*-*rs1045487* (CCCGG vs. Others)	0.03	0.77 (0.60–0.97)	0.62
Breast cancer risk	Haplotype *rs3754934*-*rs381757*-*rs10931936*-*rs1045485*-*rs1045487* (CCTGG vs. Others)	0.004	1.58 (1.16–2.14)	0.62
Breast cancer risk	Haplotype *rs12990906*-*rs3769821*-*rs6435074*-*rs3754934*-*rs3817578*-*rs10931936* (CTCCCC vs. Others)	0.011	0.71 (0.54–0.92)	0.59
Breast cancer risk	Diplotype *rs3817578*-*rs10931936*- *rs1045485* (CCG-CTG) vs. Others)	0.004	2.01 (1.25–3.22)	
Breast cancer risk	Diplotype *rs3817578*-*rs10931936*- *rs1045485* (CCC, CTG) vs. Others)	< 0.001	5.04 (2.17–11.71)	
Breast cancer risk	Diplotype *rs3754934*-*rs3817578*-*rs10931936*-*rs1045485* (CCCG-CCTG vs. Others)	0.007	1.93 (1.38–2.80)	
Breast cancer risk	Diplotype *rs3754934*-*rs381757*-*rs10931936*-*rs1045485*-*rs1045487* (CCCGG-CCTGG) vs. Others)	0.019	1.78 (1.10–2.90)	
Age of diagnosis	*rs1045487*(AA vs. GG)	0.022	0.37 (0.14–0.97)	
Age of menarche	*rs3834129* (Ins/Del vs. Ins/Ins)	0.034	0.83 (0.72–0.96)	
Age of menarche	*rs2037815* (AA vs. GG)	0.026	0.79 (0.64–0.97)	
BMI	*rs13113* (AA vs. TT)	0.029	0.92 (0.87–0.98)	
ER (Pos. vs. Neg.)	*rs3754934* (CA vs. CC)	0.008	0.40 (0.20–0.78)	
ER/PR + vs. TNBC	*rs7608692* (AA vs. GG)	0.039	1.56 (1.03–2.36)	
ER (Pos. vs. Neg.)	Haplotype rs3754934-rs3817578-rs10931936-rs1045485 (ATCG vs. Others)	< 0.001	0.25 (0.12–0.51)	0.61
BMI	Diplotype *rs12990906*- *rs3769821*-*rs6435074*-*rs3754934*-*rs3817578*-*rs10931936* (TTCCCC-TTCCCC vs. Others)	0.004	1.04 (1.02–1.07)	
Stage (Late vs. Early)	Diplotype *rs12990906*-*rs3769821*-*rs6435074*-*rs3754934*-*rs3817578*-*rs10931936 (*TTCCCC-CTCCCC vs. Others)	0.017	3.21 (1.23–8.37)	
Her2 (Pos. vs. Neg.)	Diplotype *rs6435074*-*rs3754934*-*rs3817578*-*rs10931936*-*rs1045485*-*rs1045487* (CCCCGG, ACCTGG vs. Others)	0.043	1.96 (1.02–3.78)	

^a^ The results were adjusted for BMI, age at first gestation, and Menopause status

**Table 5:** Association of CASP8 polymorphism with overall survival

Characteristics	Polymorphism	P-value _Adj_. ^a^	OR/HR (95%CI) _Adj_
Overall survival	rs3754934 (A vs. C)	0.022	0.46 (0.23–0.89)
Overall survival in Hormone receptor-positive patients	rs3754934 (A vs. C)	0.038	0.37 (0.14–0.95)
Overall survival in Hormone receptor-positive patients	rs3754934 (AA vs. CC)	0.002	0.09 (0.0.2–0.43)

^a^ The results were adjusted for BMI, age at first gestation, and Menopause status

The CTG haplotype of *rs3817578*-*rs10931936*-*rs1045485*, with a prevalence of 18.8%, among the haplotypes was associated with an increased risk of breast cancer (*p*_*Adj*_<0.001). Two 4-SNPs haplotypes, two 5-SNPs haplotypes and a 6-SNPs haplotype were also associated with the risk of breast cancer in the study population. Since the frequency of identified haplotypes with more SNPs was lower than 10%, they were not investigated in this study. Diplotypes were also identified using the haplotype data. Based on the identified diplotypes with a frequency of more than 10%, four diplotypes [*rs3817578*-*rs10931936*- *rs1045485 (*CCG-CTG*)*, *rs3817578*-*rs10931936*- *rs1045485 (*CCC, CTG*)*, *rs3754934-rs3817578-rs10931936-rs1045485 (*CCCG-CCTG*)* and *rs3754934-rs381757-rs10931936-rs1045485-rs1045487 (*CCCGG-CCTGG*)*] were associated with breast cancer risk. Significant results have been reported in Tables 4 and 5.

### Association of *CASP8* polymorphisms, haplotypes and diplotypes with clinicopathological features and overall survival

Genotypes, haplotypes, and diplotypes were extensively analyzed for a potential correlation/association with breast cancer clinicopathological characteristics and overall survival. Significant results have been presented in Tables 4 and 5.

Evaluation of the genotypes with respect to clinicopathological features specified the association of *rs3834129* (*p* = 0.034) and *rs2037815* with menstrual age (*p* = 0.026), *rs1045487* with the diagnosis age (*p* = 0.022), *rs13113* with BMI (*p* = 0.029), *rs7608692* with molecular category (*p* = 0.039) and *rs3754934* with ER status (*p* = 0.008).

Haplotype analysis identified a four-SNPs haplotype correlated with ER status (*p* < 0.001). Furthermore, three six-SNPs diplotypes were correlated with the stage of the disease (*p* = 0.017), HER2 status (*p* = 0.043), and BMI (*p* = 0.004).

**Fig. 1:**
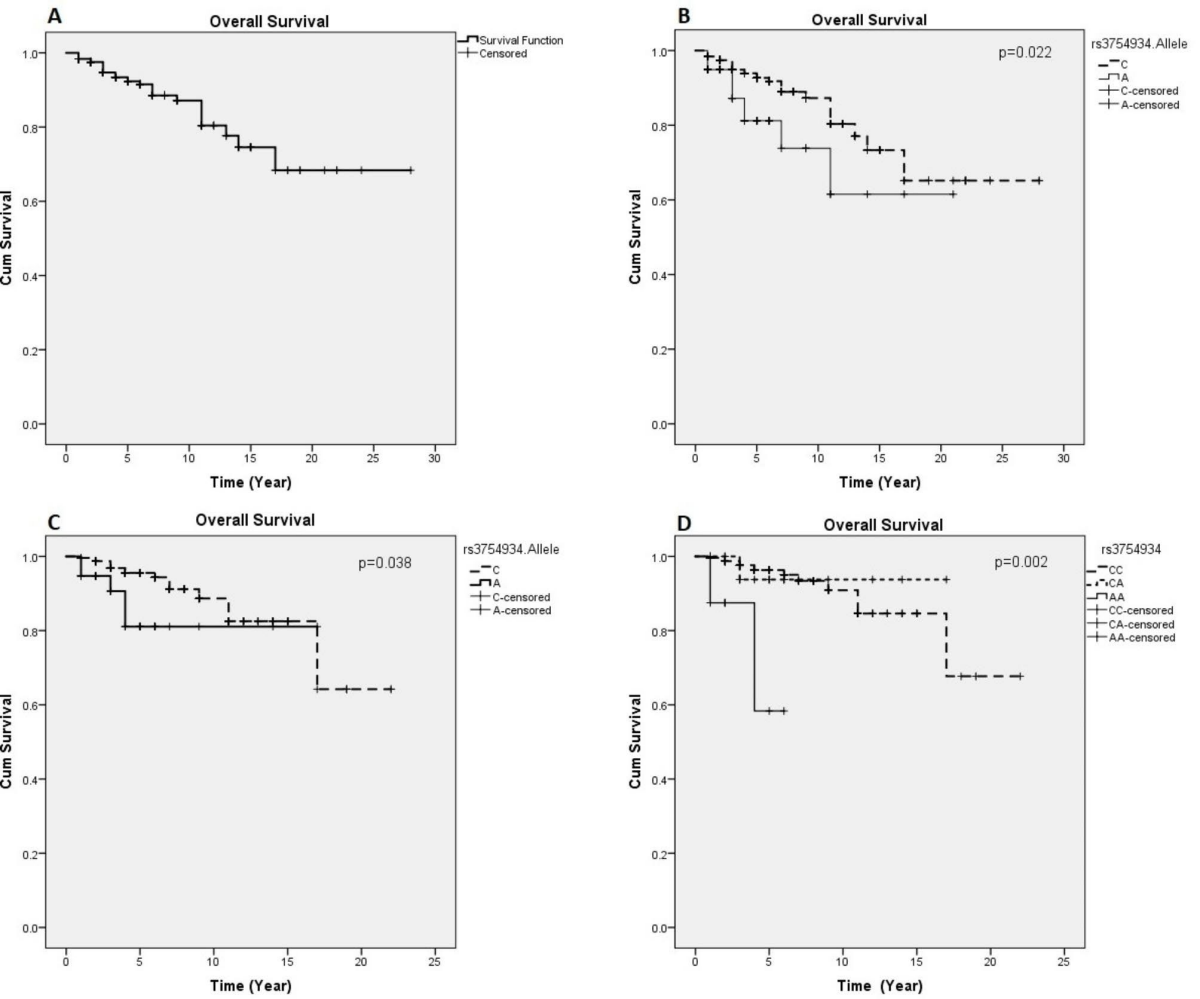
Overall Survival curves in total population (A and B) and in Hormone receptor-positive breast cancer patients (C and D) A: Kaplan-Meier overall survival curves of patients with breast cancer in total population; **B**: Kaplan-Meier overall survival curves for rs3754934 alleles (A vs. C) in all breast cancer patients; **C**: Kaplan-Meier overall survival curves for rs3754934 alleles (A vs. C) in Hormone receptor-positive breast cancer patients; **D**: Kaplan-Meier overall survival curves for rs3754934 genotypes (AA & AC vs. CC) in Hormone receptor-positive breast cancer patients

Evaluation of overall survival in patients showed that 10-year overall survival was 87% (Fig. 1A). Overall survival comparison between different genetic models of *rs3754934* polymorphism showed that the C allele was associated with a lower risk of death than the A allele [*p* = 0.022; HR = 0.46, 95% CI (0.23–0.89)] in all patients (Fig. 1B), as well as in hormone-positive group [*p* = 0.038; HR = 0.37, 95% CI (0.14–0.95)] (Fig. 1C). Furthermore, the CC genotype was associated with a lower risk of death than the AA genotype in the hormone-positive group [*p* = 0.002; HR = 0.09, 95% CI (0.02–0.43)] (Fig. 1D). However, we did not find any haplotypes and diplotypes associated with overall survival.

## Discussion

Dysregulation of apoptosis has been well known in the pathogenesis of cancer. CASP8, as a key element of apoptosis, has been represented with several genomic variations in association with breast cancer [21]. Furthermore, its overexpression can lead to induced programmed cell death in breast tumors [22, 23]. Our results indicate variations in *CASP8* are associated with the risk of breast cancer as well as clinicopathological features.

Regarding the *rs3834129*, as the most prevalent validated variant, I/D and D/D genotypes have been associated with 1.32 times and 1.42 times lower risk of breast cancer, respectively, indicating a dose-dependent effect of deletion allele similar to the reports in a Chinese population [24]. While a large study on the Europeans found no significant outcome [25], a meta-analysis has confirmed a reduced risk of breast cancer in association with the deletion allele, resulting in a reduction in the overall risk of cancer in the Asian and Caucasian populations but not in Africans [26]. Consistent with the association of *rs7698692* A-allele carriers with a 47% increased risk of the disease in the dominant model, data from a meta-analysis study showed the association of A allele with a 35% increased risk of cancer in the Asian population [27]. In addition, *rs10931936* may increase the risk of breast cancer by up to 73%, and carriers of the T allele in the dominant model also had a two-fold increased risk. In a GWAS in England, the association of *rs10931936* with breast cancer was reported with a 13% increased risk (11). This result was again confirmed by a 7% increased risk in the European population [28]. However, a study on In Situ breast cancer patients reported no association between this polymorphism and breast cancer risk [29]. While A allele carriers of *rs3754934* polymorphism in the dominant model had a 51% reduced risk of breast cancer in our population, a study of this variant in the British population did not indicate a significant association [11].

Association studies have confirmed the higher statistical power of haplotype analyses compared with alleles or genotypes analysis itself [30, 31]. In this regard, haplotype analysis indicated combinations of multiple loci of *CASP8*, including a 3-SNPs, a 4-SNPs, and a 5-SNPs haplotypes, associated with 58–78% increased risk of breast cancer in the study population. In two previous studies considering different polymorphisms of *CASP8*, several haplotypes, including *rs7608692, rs3834129, rs3817578*, and *rs1045485*, have been reported to be associated with a 28–31% increased risk of breast cancer [11, 12]. In these studies, two polymorphisms *rs3834129* and *rs1045485* have been introduced as prominent risk-related variants in line with the present study.

While a previous study has not provided such associations [11], another research has reported some *CASP8* variants related to pathological factors [32]. Considering age, associated markers may be favorable in setting up a direct-to-consumer test for early diagnosis in routine screening or assessment of prognosis. Previous findings have shown that patients diagnosed at lower ages had more aggressive features and worse prognoses than those at higher ages [33]. These results suggest that the genetic architecture of the disease may be different in older patients compared to younger, and possibly unknown genetic factors may be responsible for different tumor behaviors. However, many of the molecular mechanisms of these effects are unknown and require functional studies to identify common pathways and potential diagnostic and prognostic targets.

The importance of polymorphisms is known as prognostic markers, as polymorphisms can play a leading role in altering the uptake and absorption of chemotherapy drugs and may influence the response to chemotherapy and, ultimately, the outcome of the disease [34, 35]. However, just *CASP8 rs3754934* in the study population showed a relationship with prognosis. Previously, the association of *rs3769821* [36] and *rs1045485* [37] polymorphisms with an increased risk of death in advanced lung adenocarcinoma and breast cancer, respectively, have been reported. Also, the *rs3834129* deletion allele was associated with poor prognosis in the German population, which contradicts the protective effect of this allele in breast cancer [37].

## Conclusion

The present study with a carefully selected range of genetic markers across the *CASP8* gene region can add more evidence to the literature about the overall role of the gene in breast cancer and improve the information about the genetic basis of the disease. Based on the results of this study, which was conducted for the first time in the Northeastern female population of Iran, *CASP8* gene polymorphisms, haplotypes, and diplotypes may be used as predictive markers for the risk and prognosis of breast cancer. In addition, identified haplotypes and diplotypes which carry certain risk-related alleles may have the ability to be used in multigenic tests to calculate individual risk levels for personalized medicine purposes.

These findings, however, suggest that there is a difference in the allele frequency of considered variants in Iranian populations compared to Asian-related reports. This finding may indicate profound differences in the genetic background of populations and consequently different effects of alleles. Given that the eleven variants studied in this project were studied for the first time in Iran, highly-quality controlled frequencies obtained in this project can be used in calculating the appropriate sample size for future studies. However, identifying the mechanism of action of these haplotypes can also help to identify the tumorigenic process and may lead to opening new windows to the identification of therapeutic targets.

## Electronic supplementary material

Below is the link to the electronic supplementary material.

Supplementary Material 1

## Data Availability

The datasets generated and/or analyzed during the current study are not publicly available due Mashhad University of Medical Sciences research council rules, but are available from the corresponding author on reasonable request.
