# Supplementary material for: Genetic contribution of caspase-8 variants and haplotypes to breast cancer risk and prognosis: a case-control study in Iran
Source: BMC Med Genomics. 2023 Apr 4;16:72. doi: 10.1186/s12920-023-01484-0 (PMC10071634; doi:10.1186/s12920-023-01484-0)
Supplement: Supplementary file 1 — Supplementary Material 1 [file 12920_2023_1484_MOESM1_ESM.docx]

**Supplementary Table 1. Characteristics of the selected polymorphisms based on genome built 146**

| **rs number** | **Position** | **Heterozygosity (%)** | **Validation**  **(global)** | **MAF** | **function** | **Association(Reference)** |
| --- | --- | --- | --- | --- | --- | --- |
| [**rs3834129**](http://www.ncbi.nlm.nih.gov/projects/SNP/snp_ref.cgi?rs=3834129) | [201232809](http://www.ncbi.nlm.nih.gov/nuccore/NT_005403.18?report=graph&m=106736795&v=106736745:106736845&c=3366FF&theme=Details&flip=false&select=null&content=5&color=0&decor=0&layout=0&spacing=0) | 47.9 | 1000G | 0.396 | 5' near gene | Breast cancer(1) |
| [**rs2037815**](http://www.ncbi.nlm.nih.gov/projects/SNP/snp_ref.cgi?rs=2037815) | [201236992](http://www.ncbi.nlm.nih.gov/nuccore/NT_005403.18?report=graph&m=106740978&v=106740928:106741028&c=3366FF&theme=Details&flip=false&select=null&content=5&color=0&decor=0&layout=0&spacing=0) | 49.8 | hapmap | 0.467 | intron | multiple sclerosis(2) |
| [**rs7608692**](http://www.ncbi.nlm.nih.gov/projects/SNP/snp_ref.cgi?rs=7608692) | [201246236](http://www.ncbi.nlm.nih.gov/nuccore/NT_005403.18?report=graph&m=106750222&v=106750172:106750272&c=3366FF&theme=Details&flip=false&select=null&content=5&color=0&decor=0&layout=0&spacing=0) | 32.5 | hapmap | 0.204 | intron | Breast cancer(3) |
| [**rs12990906**](http://www.ncbi.nlm.nih.gov/projects/SNP/snp_ref.cgi?rs=12990906) | [201249901](http://www.ncbi.nlm.nih.gov/nuccore/NT_005403.18?report=graph&m=106753887&v=106753837:106753937&c=3366FF&theme=Details&flip=false&select=null&content=5&color=0&decor=0&layout=0&spacing=0) | 48.9 | hapmap | 0.425 | intron | non-small cell lung cancer(4) |
| [**rs3769821**](http://www.ncbi.nlm.nih.gov/projects/SNP/snp_ref.cgi?rs=3769821) | [201258707](http://www.ncbi.nlm.nih.gov/nuccore/NT_005403.18?report=graph&m=106762693&v=106762643:106762743&c=3366FF&theme=Details&flip=false&select=null&content=5&color=0&decor=0&layout=0&spacing=0) | 48.6 | hapmap | 0.416 | intron | Breast cancer(1) |
| [**rs6435074**](http://www.ncbi.nlm.nih.gov/projects/SNP/snp_ref.cgi?rs=6435074) | [201263224](http://www.ncbi.nlm.nih.gov/nuccore/NT_005403.18?report=graph&m=106767210&v=106767160:106767260&c=3366FF&theme=Details&flip=false&select=null&content=5&color=0&decor=0&layout=0&spacing=0) | 42.2 | 1000G | 0.302 | intron | Breast cancer(5) |
| [**rs3754934**](http://www.ncbi.nlm.nih.gov/projects/SNP/snp_ref.cgi?rs=3754934) | [201267384](http://www.ncbi.nlm.nih.gov/nuccore/NT_005403.18?report=graph&m=106771370&v=106771320:106771420&c=3366FF&theme=Details&flip=false&select=null&content=5&color=0&decor=0&layout=0&spacing=0) | 25.7 | hapmap | 0.152 | intron | Breast cancer(6) |
| [**rs3817578**](http://www.ncbi.nlm.nih.gov/projects/SNP/snp_ref.cgi?rs=3817578) | [201271872](http://www.ncbi.nlm.nih.gov/nuccore/NT_005403.18?report=graph&m=106775858&v=106775808:106775908&c=3366FF&theme=Details&flip=false&select=null&content=5&color=0&decor=0&layout=0&spacing=0) | 24.2 | hapmap | 0.141 | intron | Breast cancer(7) |
| [**rs10931936**](http://www.ncbi.nlm.nih.gov/projects/SNP/snp_ref.cgi?rs=10931936) | [201279205](http://www.ncbi.nlm.nih.gov/nuccore/NT_005403.18?report=graph&m=106783191&v=106783141:106783241&c=3366FF&theme=Details&flip=false&select=null&content=5&color=0&decor=0&layout=0&spacing=0) | 42.7 | hapmap | 0.31 | intron | Breast cancer(1) |
| [**rs1045485**](http://www.ncbi.nlm.nih.gov/projects/SNP/snp_ref.cgi?rs=1045485) | [201284866](http://www.ncbi.nlm.nih.gov/nuccore/NT_005403.18?report=graph&m=106788852&v=106788802:106788902&c=3366FF&theme=Details&flip=false&select=null&content=5&color=0&decor=0&layout=0&spacing=0) | 12.7 | hapmap | 0.068 | Exon (missense) | Breast cancer(1) |
| [**rs1045487**](http://www.ncbi.nlm.nih.gov/projects/SNP/snp_ref.cgi?rs=1045487) | [201284973](http://www.ncbi.nlm.nih.gov/nuccore/NT_005403.18?report=graph&m=106788959&v=106788909:106789009&c=3366FF&theme=Details&flip=false&select=null&content=5&color=0&decor=0&layout=0&spacing=0) | 25.4 | hapmap | 0.149 | Exon (synonymous) | Breast cancer(3) |
| [**rs13113**](http://www.ncbi.nlm.nih.gov/projects/SNP/snp_ref.cgi?rs=13113) | [201287439](http://www.ncbi.nlm.nih.gov/nuccore/NT_005403.18?report=graph&m=106791425&v=106791375:106791475&c=3366FF&theme=Details&flip=false&select=null&content=5&color=0&decor=0&layout=0&spacing=0) | 44.9 | 1000G | 0.341 | 3' UTR | Breast cancer(3) |

**Supplementary Table 2. The sequence of primers used for genotyping**

| **Polymorphism** | **Primer name** | **Sequence** | **Amplification method** |
| --- | --- | --- | --- |
| **rs3834129** | Outer Forward | 5' AGTGAAAACTTCTCCCATGGCCTC 3' | ARMS-PCR |
|  | Outer Reverse | 5' GATTGATACTGGCACAGTATACTTACC 3' |  |
|  | Inner Forward | 5' GTAATTCTTGCTCTGCCAAGCTG 3' |  |
|  | Inner Reverse | 5' CCAAGGTCACGCAGCTAGTAAG 3' |  |
| **rs2037815** | Common Forward | 5' TGGAGTTTCATCATGTTTCC 3' | AS-PCR |
|  | Reverse G | 5' TATGACAAAGAGAATCAGAGACTTC 3' |  |
|  | Reverse A | 5' TATGACAAAGAGAATCAGAGACTTT 3' |  |
|  | Forward | 5' GGCTCAAGCTATCCATCTGC 3' | Sequencing |
|  | Reverse | 5' CTGGGGAAAAGGAACAAACA 3' |  |
| **rs7608692** | Outer Forward | 5' CCAGGTTCAAGCAATTCTCCTGC 3' | AS-PCR |
|  | Outer Reverse | 5' CTGGGAATAAATCAACAGACAATCACACA 3' |  |
|  | Inner Forward | 5' CCTCTTTCGTTTTCAGTCTGGCATTTA 3' |  |
|  | Inner Reverse | 5' CCCTGGGCTGTCTTTTTATTCCAAC 3' |  |
| **rs12990906** | Outer Forward | 5' TTCAATCGGGTTGTTTTCTTATTG 3' | ARMS-PCR |
|  | Outer Reverse | 5' CCAGCGGACTACACTTACTTCAAA 3' |  |
|  | Inner Forward | 5' GGCAAATCTGTTTTCCTGCC 3' |  |
|  | Inner Reverse | 5' TTCTCAGCCATGAACTTAGAAGGTTA 3' |  |
| **rs3769821** | Forward | 5' GGAGAGTCCAGAAGACTTTATAGATC 3' | RFLP-PCR |
|  | Reverse | 5' GGGTGGAGAGATAAAAGGAAAC 3' |  |
|  | Forward | 5' CGAATCCACATCAAAACATTAGAG 3' | Sequencing |
|  | Reverse | 5' ACTTTTTCACTCTGAGCAGTCTCC 3' |  |
| **rs6435074** | Forward | 5' AAGAGCCTCAAAAAGGTTT 3' | HRM |
|  | Reverse | 5' TTAGAATAATTTCCTAAAAGTAGTAT 3' |  |
|  | Forward | 5' TATGG GGACGGAGCTATGC 3' | Sequencing |
|  | Reverse | 5' CCACCATCCAAATATAAATGCCC 3' |  |
| **rs3754934** | Outer Forward | 5' AGTTGTAACTGAATGGGGTTTCTCTCTC 3' | ARMS-PCR |
|  | Outer Reverse | 5' CCTCTCTTATCTTGATGCCTCTCGATA T 3' |  |
|  | Inner Forward | 5' TTACAGCTCCGTAAAGA CTTAGTCA CCT 3' |  |
|  | Inner Reverse | 5' AGGGTTTTCTGCCACACTTAAAGATTC 3' |  |
| **rs3817578** | Outer Forward | 5' CAGTGATACACACACAGGGGCATAA 3' | ARMS-PCR |
|  | Outer Reverse | 5' CATTTCTGAGACCAAAGAGAGGGGT 3' |  |
|  | Inner Forward | 5' GCAGTCTTTCCCAGCTCTGACG 3' |  |
|  | Inner Reverse | 5' GTGCAGATGCAGCCCAGAACT 3' |  |
| **rs10931936** | Outer Forward | 5' TTGAAAAAAAAGAGTCGAGGTAATTGAC 3' | ARMS-PCR |
|  | Outer Reverse | 5' AGGTTTTCTTCAGTCTCTCTGTGTTCAG 3' |  |
|  | Inner Forward | 5' AAGCCTATACAATCCTCTGATTCATACATC 3' |  |
|  | Inner Reverse | 5' AAGAATAGTTGCTGGCTCTATGAGATGA 3' |  |
| **rs1045485** | Forward | 5' CAT TTTTGAGATCAAGCCCCGC 3' | RFLP-PCR |
|  | Reverse | 5' CCCTTGTCTCCATGGGAGAGGA 3' |  |
|  | Forward | 5' CTCCAGCTGTGGTCTGTGAA 3' | Sequencing |
|  | Reverse | 5' TCCATGGGAGAGGATACAGC 3' |  |
| **rs1045487** | Forward | 5' ATCTGCTGTATCCTCTCCC 3' | HRM |
|  | Reverse | 5' TCCTGTCCATCAGTGCCA 3' |  |
|  | Forward | 5' TTGAGATCAAGCCCCACGAT 3' | Sequencing |
|  | Reverse | 5' GTCAGCCTCATCCGGGATAT 3' |  |
| **rs13113** | [VIC/FAM] Probe | TTAATAAAACAAAATTTGTTTGAAA[A/T]CTTTTAAAAATTCAAATGATTTTTA | TaqMan-PCR |

**Supplementary Table 3. Amplification reactions**

| **Supplementary Table 3a. Amplification reactions for ARMS method** | | | | | |
| --- | --- | --- | --- | --- | --- |
| Polymorphism | **rs3834129** | **rs12990906** | **rs3754934** | **rs3817578** | **rs10931936** |
| Material | Volume (µl) | Volume (µl) | Volume (µl) | Volume (µl) | Volume (µl) |
| Master mix | 4 | 4 | 4 | 4 | 4 |
| Outer Forward | 1 (100 pM) | 1.1 µl (100 pM) | 1 l (100 pM) | 1.1 (100 pM) | 1.1 (100 pM) |
| Outer Reverse | 1 (100 pM) | 1.1 µl (100 pM) | 1 (100 pM) | 1.1 (100 pM) | 1.1 (100 pM) |
| Inner Forward | 1.3 (130 pM) | 1.5 (136 pM) | 1.2 (120 pM) | 1.2 (110 pM) | 1.5 (136 pM) |
| Inner Reverse | 1.2 (120 pM) | 1.5 (136 pM) | 1.3 (130 pM) | 1.5 (136 pM) | 1.5 (1136 pM) |
| Template DNA | 1.5 (150 ng) | 1.5 µl (150 ng) | 1.5 (150 ng) | 1.5 (150 ng) | 1.5 (150 ng) |
| Water | 0 | 0.3 | 0 | 0.6 | 0.3 |
| Total volume | 10 µl | 11 µl | 10 µl | 11 µl | 11 µl |

| **Supplementary Table 3b. Amplification reactions for AS-PCR method** | | |
| --- | --- | --- |
| Polymorphism | **rs2037815** | **rs7608692** |
| Material | Volume (µl) | Volume (µl) |
| Master mix | 4 | 4µl |
| Common Forward | 1 (100 pM) | 1 (100 pM) |
| Reverse | 1 (100 pM) | 1 (100 pM) |
| Template DNA | 1.5 (150 ng) | 1.5 (150 ng) |
| Water | 2.5 | 2.5 |
| Total volume | 10 µl | 10 µl |

| **Supplementary Table 3c. Amplification reactions for RFLP method** | | |
| --- | --- | --- |
| Polymorphism | **rs3769821** | **rs1045485** |
| Material | Volume (µl) | Volume (µl) |
| Master mix | 7 | 7 |
| Common Forward | 1.5 (100 pM) | 1.5 (100 pM) |
| Reverse | 1.5 (100 pM) | 1.5 (100 pM) |
| Template DNA | 2 (200 ng) | 2 (200 ng) |
| Water | 3 | 3 |
| Total volume | 15 µl | 15 µl |

| **Supplementary Table 3d. Amplification reactions for HRM method** | | |
| --- | --- | --- |
| Polymorphism | **rs6435074** | **rs1045487** |
| Material | Volume (µl) | Volume (µl) |
| Master mix | 2.5 | 2.5 |
| Common Forward | 2 (100 pM) | 2 (100 pM) |
| Reverse | 2 (100 pM) | 2 (100 pM) |
| Template DNA | 1 (100 ng) | 1 (100 ng) |
| Water | 12.5 | 12.5 |
| Total volume | 20 µl | 20 µl |

| **Supplementary Table 3e. Amplification reactions for TaqMan method** | |
| --- | --- |
| Polymorphism | **rs13113** |
| Material | Volume (µl) |
| TaqMan Universal PCR Master mix | 3 |
| 20x SNP Genotyping AssayMix | 0.12 |
| Template DNA | 1 (50 ng) |
| Water | 8.38 |
| Total volume | 12.5 µl |

**Supplementary Table 4. Amplification protocols**

| **Supplementary Table 4a. Amplification protocols for ARMS method** | | | | | | | | | | | |
| --- | --- | --- | --- | --- | --- | --- | --- | --- | --- | --- | --- |
| **Polymorphism** | | **rs3834129** | | **rs12990906** | | **rs3754934** | | **rs3817578** | | **rs10931936** | |
| **Stage** | | Temp ºC | Time | Temp ºC | Time | Temp ºC | Time | Temp ºC | Time | Temp ºC | Time |
| **Primary denature** | | 95 | 5 min | 95 | 5 min | 95 | 5 min | 95 | 5 min | 95 | 5 min |
| **35 cycles** | Denature | 95 | 30 s | 95 | 30 s | 95 | 30 s | 95 | 30 s | 95 | 30 s |
|  | Annealing | 58 | 30 s | 58 | 30 s | 66 | 20 s | 58 | 30 s | 63 | 30 s |
|  | Extension | 72 | 30 s | 72 | 30 s | 72 | 25 s | 72 | 30 s | 72 | 30 s |
| **Final extension** | | 72 | 7 min | 72 | 7 min | 72 | 7 min | 72 | 7 min | 72 | 7 min |

| **Supplementary Table 4b. Amplification protocols for AS-PCR method** | | | | | |
| --- | --- | --- | --- | --- | --- |
| **Polymorphism** | | **rs2037815** | | **rs7608692** | |
| **Stage** | | **Temp ºC** | **Time** | **Temp ºC** | **Time** |
| **Primary denature** | | 95 | 5 min | 95 | 5 min |
| **30 cycles** | Denature | 95 | 15 s | 95 | 15 s |
|  | Annealing | 57 | 15 s | 62 | 15 s |
|  | Extension | 72 | 20 s | 72 | 20 s |
| **Final extension** | | 72 | 5 min | 72 | 5 min |

| **Supplementary Table 4c. Amplification protocols for RFLP method** | | | | | |
| --- | --- | --- | --- | --- | --- |
| **Polymorphism** | | **rs3769821** | | **rs1045485** | |
| **Stage** | | **Temp ºC** | **Time** | **Temp ºC** | **Time** |
| **Primary denature** | | 95 | 5 min | 95 | 5 min |
| **30 cycles** | Denature | 95 | 15 s | 95 | 15 s |
|  | Annealing | 57 | 15 s | 62 | 15 s |
|  | Extension | 72 | 15 s | 72 | 15 s |
| **Final extension** | | 72 | 7 min | 72 | 7 min |

| **Supplementary Table 4d. Amplification protocols for HRM method** | | | | | |
| --- | --- | --- | --- | --- | --- |
| **Polymorphism** | | **rs6435074** | | **rs1045487** | |
| **Stage** | | **Temp ºC** | **Time** | **Temp ºC** | **Time** |
| **Primary denature** | | 95 | 5 min | 95 | 5 min |
| **30 cycles** | Denature | 95 | 15 s | 95 | 15 s |
|  | Annealing | 57 | 15 s | 57 | 15 s |
|  | Extension | 72 | 15 s | 72 | 15 s |
| **Final extension** | | 72 | 7 min | 72 | 7 min |
|  |  |  |  |  |  |

| **Supplementary Table 4e. Amplification protocols for TaqMan method** | | | |
| --- | --- | --- | --- |
| **Polymorphism** | | **rs13113** | |
| **Stage** | | **Temp (ºC)** | **Time** |
| **Primary denature** | | 95 | 10 min |
| **40 cycles** | Denature | 95 | 15 s |
|  | Annealing & Extension | 60 | 1 min |
| **Post read** | | 60 | 30 s |

**Supplementary Table 5. Minor allele frequencies among breast cancer patients and healthy controls and the HWE *P*-value of *CASP8* gene polymorphisms**

| **SNP ID** | **Breast cancer cases** | | | **Healthy controls** | | |
| --- | --- | --- | --- | --- | --- | --- |
|  | **MA**^a^ | **MAF^a^** | **HWE *P*-value^c, d^** | **MA** | **MAF** | **HWE *P*-value^c^** |
| rs3834129 | Del^e^ | 0.33 | 0.27 | Del | 0.41 | 0.89 |
| rs2037815 | A | 0.47 | ≤ 0.05 | G | 0.49 | 0.15 |
| rs7608692 | A | 0.41 | 0.82 | A | 0.35 | ≤ 0.05 |
| rs12990906 | C | 0.37 | 0.36 | C | 0.42 | 0.19 |
| rs3769821 | C | 0.23 | 0.26 | C | 0.22 | ≤ 0.05 |
| rs6435074 | A | 0.29 | 0.79 | A | 0.27 | 0.99 |
| rs3754934 | A | 0.09 | ≤ 0.05 | A | 0.09 | ≤ 0.05 |
| rs3817578 | T | 0.15 | 0.73 | T | 0.16 | 0.83 |
| rs10931936 | T | 0.27 | 0.56 | T | 0.17 | ≤ 0.05 |
| rs1045485 | C | 0.21 | ≤ 0.05 | C | 0.23 | ≤ 0.05 |
| rs1045487 | A | 0.16 | 0.12 | A | 0.15 | 0.75 |
| rs13113 | A | 0.4 | 0.54 | A | 0.36 | 0.92 |
| ^a^ Minor allele | | | | | | |
| ^b^ Minor allele frequency | | | | | | |
| ^c^ In order to verify the results of the genotyping, 5% of samples were randomly regenotyped for SNPs with HWE *p*≤0.05 and the results were consistent with the previously genotyped samples. | | | | | | |
| ^d^ HWE; Hardy-Weinberg Equilibrium | | | | | | |
| ^e^ Deletion | | | | | | |

**Supplementary Table 6. Genetic association analysis for the *CASP8* gene polymorphisms using different genetic models**

| **SNP ID** | **Genetic model** | **P-value _Adj._^a^** | **OR (95%CI) _Adj._** |
| --- | --- | --- | --- |
| **rs3834129** | Dominant (II+ID vs. DD) | **0.034** | 1.76 (1.04-2.97) |
|  | Recessive (ID+DD vs. II) | **0.014** | 1.58 (1.09-2.67) |
|  | Additive (II+ DD vs. ID) | 0.054 | 1.28(0.99-1.64) |
| **rs2037815** | Dominant (GA+GG vs. AA) | **0.031** | 1.44 (1.03-2.01) |
|  | Recessive (AA+GA vs. GG) | 0.809 | 0.95 (0.64-1.42) |
|  | Additive(AA+ GG vs. GA) | 0.25 | 0.86(0.67-1.11) |
| **rs7608692** | Dominant (GA + AA vs.GG) | **0.006** | 1.47 (1.12-1.93) |
|  | Recessive (GG+GA vs. AA) | 0.715 | 0.92 (0.58-1.45) |
|  | Additive (GG+ AA vs. GA) | **0.01** | 0.72(0.56-0.92) |
| **rs12990906** | Dominant (TC+TT vs. CC) | 0.388 | 1.21 (0.78-1.88) |
|  | Recessive(CC+TC vs. TT) | 0.127 | 1.32 (0.92-1.89) |
|  | Additive(CC+ TT vs. TC) | 0.68 | 1.05(0.82-1.35) |
| **rs3769821** | Dominant (TC+CC vs. TT) | 0.44 | 1.49 (0.81-1.63) |
|  | Recessive (TT+TC vs. CC) | 0.948 | 0.98 (0.52-1.83) |
|  | Additive(TT+ CC vs. TC) | **0.02** | 0.71(0.54-0.94) |
| **rs6435074** | Dominant (CA+AA vs. CC) | 0.826 | 1.04 (0.74-1.46) |
|  | Recessive (CC+CA vs. AA) | 0.873 | 1.05 (0.58-1.91) |
|  | Additive(CC+ AA vs. CA) | 0.79 | 0.96(0.75-1.24) |
| **rs3754934** | Dominant (CA+AA vs. CC) | **0.004** | 0.49 (0.27-0.78) |
|  | Recessive (CC+CA vs. AA) | 0.99 | 1.01 (0.29-3.46) |
|  | Additive(CC+ AA vs. CA) | 0.29 | 1.22(0.84-1.79) |
| **rs3817578** | Dominant (CT+CC vs. TT) | 0.55 | 1.12 (0.77-1.65) |
|  | Recessive (TT+CT vs. CC) | 0.922 | 1.06 (0.33-3.35) |
|  | Additive(TT+ CC vs. CT) | 0.78 | 0.96(0.72-1.27) |
| **rs10931936** | Dominant (CT+TT vs. CC) | **<0.001** | 2.06 (1.44-2.93) |
|  | Recessive (CC+CT vs. TT) | 0.933 | 1.03 (0.55-1.94) |
|  | Additive(CC+ TT vs. CT) | **<0.001** | 0.47(0.36-0.62) |
| **rs1045485** | Dominant (GC+GG vs. CC) | 0.25 | 1.47 (0.76-2.84) |
|  | Recessive (CC+GC vs. GG) | 0.867 | 1.03 (0.72-1.48) |
|  | Additive(CC+ GG vs. GC) | 0.36 | 0.88(0.66-1.16) |
| **rs1045487** | Dominant (GA+AA vs. GG) | 0.574 | 1.12 (0.76-1.64) |
|  | Recessive (GG+GA vs. AA) | 0.114 | 2.29 (0.82-6.37) |
|  | Additive(GG+ AA vs. GA) | 0.92 | 0.98(0.73-1.31) |
| **rs13113** | Dominant (TA+AA vs. TT) | 0.567 | 1.11 (0.78-1.57) |
|  | Recessive (TT+TA vs. AA) | 0.29 | 1.30 (0.80-2.10) |
|  | Additive(TT+ AA vs. TA) | 0.96 | 0.99(0.77-1.27) |
| ^a^ Significant data has been shown in bold. | | | |

1. Hashemi M, Aftabi S, Moazeni-Roodi A, Sarani H, Wiechec E, Ghavami S. Association of CASP8 polymorphisms and cancer susceptibility: A meta-analysis. Eur J Pharmacol. 2020;881:173201. doi: 10.1016/j.ejphar.2020.173201. PubMed PMID: 32442541.

2. Camiña-Tato M, Fernández M, Morcillo-Suárez C, Navarro A, Julià E, Edo MC, et al. Genetic association of CASP8 polymorphisms with primary progressive multiple sclerosis. J Neuroimmunol. 2010;222:70-5. doi: 10.1016/j.jneuroim.2010.03.003. PubMed PMID: 20363033.

3. Shephard ND, Abo R, Rigas SH, Frank B, Lin WY, Brock IW, et al. A breast cancer risk haplotype in the caspase-8 gene. Cancer Res. 2009;69:2724-8. doi: 10.1158/0008-5472.can-08-4266. PubMed PMID: 19318553; PubMed Central PMCID: PMCPMC2730164.

4. Rosell R, Wei J. Single nucleotide polymorphisms (SNPs) in non-small cell lung cancer (NSCLC) patients. Oncologist. 2012;17:1484-5. doi: 10.1634/theoncologist.2012-0205. PubMed PMID: 23015663; PubMed Central PMCID: PMCPMC3528379.

5. Zhang B, Beeghly-Fadiel A, Long J, Zheng W. Genetic variants associated with breast-cancer risk: comprehensive research synopsis, meta-analysis, and epidemiological evidence. Lancet Oncol. 2011;12:477-88. doi: 10.1016/s1470-2045(11)70076-6. PubMed PMID: 21514219; PubMed Central PMCID: PMCPMC3114278.

6. Pasdar A, Afzaljavan F, Shandiz FH, Kooshyar MM. 385P - Identifying CASP8 polymorphisms associated with breast cancer risk in an Iranian population. Annals of Oncology. 2019;30:ix129. doi: <https://doi.org/10.1093/annonc/mdz431.022>.

7. Camp NJ, Parry M, Knight S, Abo R, Elliott G, Rigas SH, et al. Fine-mapping CASP8 risk variants in breast cancer. Cancer Epidemiol Biomarkers Prev. 2012;21:176-81. doi: 10.1158/1055-9965.epi-11-0845. PubMed PMID: 22056502; PubMed Central PMCID: PMCPMC3253962.
